# Coeliac Disease in the 21st Century: No Longer “Kids’ Stuff”

**DOI:** 10.4021/gr376e

**Published:** 2011-11-20

**Authors:** Alfredo J. Lucendo, Álvaro García-Manzanares, Ángel Arias, Dolores Fuentes, Noemí Álvarez, Isabel Pérez, Danila Guagnozzi, Luis Rodrigo

**Affiliations:** a Department of Gastroenterology, Hospital General de Tomelloso, Tomelloso, Ciudad Real, Spain; b Department of Endocrinology and Nutrition, Hospital General de Tomelloso, Tomelloso, Ciudad Real, Spain; c Research Unit, Hospital General La Mancha Centro, Alcázar de San Juan, Ciudad Real, Spain; d Department of Gastroenterology. Hospital Universitario Central de Asturias. Oviedo, Spain

**Keywords:** Celiac disease, Adult patient, Anti-tissue transglutaminase antibodies: gluten enteropathy

## Abstract

**Background:**

We aimed to determine if Coeliac disease (CD) can be still be considered a predominantly paediatric disorder, in spite of the increased incidence of adult-onset CD reported in recent years.

**Methods:**

An observational, descriptive, and retrospective study was developed at two Spanish hospitals. Data was collected and analyzed from all paediatric and adult patients newly diagnosed with CD throughout the year 2010. CD diagnoses were based on a concordant clinical history, serology, HLA-DQ compatibility, the presence of mucosal lesions in duodenal biopsies with gluten dependence of symptoms, and histological lesions.

**Results:**

A total of 79 patients were diagnosed with CD throughout 2010, of which 68 (86.1%) were adults. Classic symptoms (diarrhoea and iron-deficiency anaemia) were more frequent in children (90.9%), being present in only 54.4% of adults (p = 0.02). Adult patients showed, mainly, abdominal pain, dyspepsia, and GERD-related symptoms. Villous atrophy (Marsh III) was present in 63.7% of children, but only in 19.1% of adults (p = 0.004). Positive tTGA was present in 81.8% of the children and only in 19.1% of the adults (p = 0.004). Haemoglobin levels were significantly lower in children (p = 0.025), but no differences were observed in iron and ferritin blood levels.

**Conclusions:**

Our study shows that adult-onset CD was the predominant presentation in two hospitals in Spain in the year 2010. Therefore, CD can no longer be considered a predominantly paediatric disorder. Marsh I and negative tTGA titters are characteristic in most of adults. New diagnostic algorithms are needed to improve correct diagnosis of CD in adults.

## Introduction

Coeliac disease (CD) is a chronic disorder that primarily affects the digestive system. It is characterized by the presence of inflammatory changes in the small bowel that are triggered and maintained by an immunological response provoked by the exposure to gluten in the diet [1]. The ingestion of food containing gluten gives rise to different types of lesions in the small bowel mucosa [2, 3] of genetically susceptible individuals, sometimes leading to various associated disorders. CD constitutes one of the main causes of malabsorption in developed countries [4].

Up until twenty years ago, CD was considered to be present predominantly in children and with a low prevalence. However, in recent years several epidemiological studies have clearly shown an increasing number of diagnosed cases in both children and adults. It is especially noteworthy that adult-onset CD has been increasingly described in last few years with continuous growth in incidence rates over time [5-9] and a considerable proportion of cases being diagnosed in the elderly [10]. As a consequence, CD today constitutes a very prevalent disease affecting between 1 - 3% of the European and US populations at some stage in life [11].

Typical manifestations of CD are characterized by severe symptoms of malabsorption (diarrhoea, steatorrhoea, growth retardation, and nutritional deficiencies); indeed, this constitutes the classic manifestation predominant in children. Generally, however, CD in adults clinically presents mild and non-specific symptoms, with digestive complaint being either absent or of secondary importance [12]. It is thus common for adult patients to go undiagnosed for many years [13]. It has been estimated that for every new patient diagnosed with CD, 2 to 10 cases may go undiagnosed. The average period of evolution of symptoms in adults prior to being diagnosed is estimated to be up to 17 years [6].

This paper analyzes all new patients diagnosed with CD at two Spanish hospitals for the entire year of 2010. We compared the clinical, serological, genetic, and histological characteristics and their age distribution in order to determine whether CD is still predominantly a paediatric disorder.

## Methods

All newly diagnosed CD patients at two general hospitals in different Spanish regions throughout the year 2010 (#1 Tomelloso General Hospital and #2 Central University Hospital in Asturias) were analyzed. The patient search was carried out by reviewing the databases of the departments of gastroenterology, paediatrics, pathology, and the clinical laboratories of both hospitals in order to ensure that all cases were analyzed. A patient was considered to be an adult if he or she was aged 16 or over.

Diagnosis of CD was based on the following five basic criteria: 1) Presence of a concordant gluten enteropathy in duodenal biopsies ranging from stage I (increased density of intraepithelial lymphocytes > 25%) to stage III (villous atrophy), classified in accordance with the system proposed by Michael Marsh in 1992 [3]. A minimum of six samples were taken with the aid of a standard needle endoscopic jumbo-type forceps, on the second and/or third duodenal portions, and analyzed by board certified pathologists from each hospital. Duodenal biopsies were repeated in all those patients exhibiting a Marsh I stage 6 months after setting up a strict gluten-free diet (GFD) in order to confirm the resolution of lymphocytic infiltrate, differentiating CD from gluten sensitivity [14, 15]. 2) Existence of a compatible clinical picture, including digestive and extra-gastrointestinal symptoms. 3) Positive immunoglobulin (IgA) anti-tissue transglutaminase antibody (tTGA) titters, determined by ELISA tests. The positivity threshold was established at 2 U/mL. In cases of IgA deficiency, IgG tTGA was determined. 4) Presence of an HLA-DQA1*05-DQB1*02 (DQ2) or HLA- DQA1*03-DQB1*0302 (DQ8) haplotype, which confers risk for CD. Gene analyses were developed in both centres by polymerase chain reaction (PCR)-based typing techniques from EDTA-anticoagulated blood. 5) Clinical, histopathological, and biochemical recovery after the initiation of a GFD. Those patients who only exhibited improvement of symptoms after GFD without evidence of histological and analytical recovery were given the diagnosis of gluten sensitivity and were not included in this study.

Since no single test can detect the early stages of celiac disease without atrophy, a combination of clinical history, positive serology, HLA DQ compatibility, and gluten dependence of symptoms and histological lesions was used to achieve the CD diagnosis.

Data on the following parameters were also collected: sex, age at diagnosis, family history of CD, main and associated symptoms, duration of evolution prior to diagnosis, HLA haplotype conferring risk for CD, existence of autoimmune associated diseases, IgA anti-tissue transglutaminase antibody (tTGA) titters, serum iron and ferritin levels, body mass index (BMI) for adults, and weight and height percentiles for children. Classic CD symptoms were considered if patients presented primary digestive complaints in the form of diarrhoea or iron-deficiency anaemia which, with the related signs of malabsorption, is considered to be the most usual clinical presentation of CD [16-18].

Quantitative data distribution was expressed as mean and standard deviation. Sociodemographic and clinical data were compared in adult and paediatric patients with the aid of the Chi squared test (or the Fisher exact test, where appropriate). Levels of tTGA in relation to the Marsh scale were explored with a non-parametric Kruskal-Wallis test while comparisons between groups were carried out with the U Mann-Whitney test with Bonferroni correction for multiple comparisons.

Statistical analyses were performed with the aid of PASW 18.0 statistical analysis software (SPSS Inc).

This research was carried out in accordance with the Helsinki Declaration and was approved by the research ethics committees of our respective institutions.

## Results

**Figure 1:**
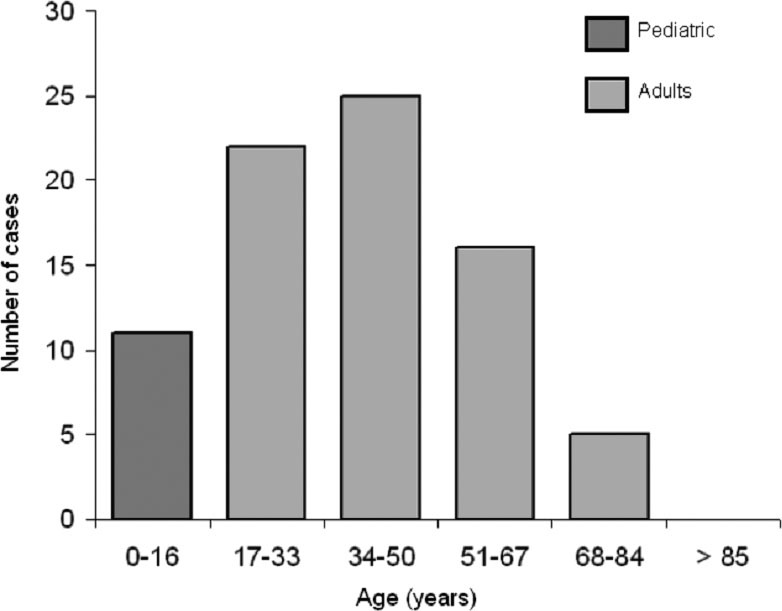
Age distribution of all celiac patients diagnosed in 2010 at two Spanish Hospitals; results show that CD predominates in adult patients between the *3rd and 5th decades of life*.

Throughout 2010, a total of 79 new patients were diagnosed with CD (25 cases in Hospital #1 and 54 cases in Hospital #2), of which 68 (86.1%) were older than 16 year-old. The proportion between CD in adults and children was 6.18/1. Adult-onset CD occurred predominantly in the 3rd to 5th decades of life (Fig 1).

No significant differences were detected between patients from the two hospitals regarding demographic, biochemical, immunological, and genetic characteristics, except for the higher proportion of females with adult-onset CD in Hospital #1 and the lower haemoglobin and serum iron levels in Hospital #2 (Table 1).

**Table 1:** Comparative Characteristics of All Cases of Celiac Disease Diagnosed During the Year 2010 in Two General Hospitals in Spain. Data are Alternatively Expressed as Mean ± Standard Deviation, or Number of Patient and Percentage

	Adult CD patients			Paediatric CD patients		
	Hospital #1	Hospital #2	p	Hospital #1	Hospital #2	p
Number or patients (%)	20 (80%)	48 (88%)	0.29	5 (20%)	6 (11%)	0.29
Female / Male (%)	19/1 (95%)	31/17 (64.58%)	0.01	4/1 (80%)	3/3 (50%)	0.30
Mean age ± SD (years)	43.50 (13.89)	43.15 (15.90)	0.93	4.03 (5.02)	3.33 (2.94)	0.77
Positive tTGA (%)	5 (25%)	8 (16.7%)	0.50	4 (80%)	5 (83.3%)	0.89
BMI (in adults)	24.95 (6.38)	24.56 (3.62)	0.76	-	-	-
Diagnostic delay	127.53 (155.11)	52.90 (53.74)	0.39	15.20 (18.62)	7 (3.46)	0.66
Classic symptoms (%)	11 (55%)	26 (54.16%)	0.95	4 (80%)	6 (100%)	0.46
Haemoglobin levels (g/dL)	13.11 (1.52)	12.971 (2.19)	0.89	12.90 (0.85)	11.283 (0.96)	0.03
Serum Iron (µg/dL)	74.30 (29.95)	71.38 (50.76)	0.31	79.50 (24.20)	44 (7.92)	0.038
Ferritin (ng/mL)	62 (114.39)	49.40 (52.96)	0.65	25.20 (12.91)	20.83 (7.28)	0.66
Familiar association (%)	3 (15%)	6 (12.5%)	0.95	0 (0%)	3 (50%)	0.24
Marsh I	13 (65%)	32 (66.7%)	0.89	1 (20%)	0 (0%)	0.25
Marsh II	3 (15%)	7 (14.6%)	1	2 (40%)	1 (16.7%)	0.55
Marsh III (a, b, c)	4 (20%)	9 (18.7%)	1	2 (40%)	5 (83.3%)	0.24
DQ2 haplotype	15 (75%)	43 (89.6%)	0.14	4 (80%)	5 (83.3%)	1
DQ8 haplotype	2 (10%)	2 (4.2%)	0.58	1 (20%)	1 (16.7%)	1
Non DQ2/DQ8	3 (15%)	3 (6.2%)	0.35	0 (0%)	0 (0%)	1

tTGA: anti-tissue transglutaminase antibodies; BMI: body mass index.

CD predominantly affected females in both children and adults, with no differences between the two groups with respect to the existence of other relatives with CD. Furthermore, the distribution of the genetic markers HLA-DQ2 and DQ8 alleles, which predispose individuals to developing CD, showed no differences between age groups.

That being said, several relevant differences were found between paediatric and adult- onset CD (Table 2). The classic symptoms (diarrhoea and iron-deficiency anaemia) were the most frequent forms of presentation in children (90.9%) whereas they were present in only half of the adults (54.4%; p = 0.02). In contrast, adult patients had a wider complex of symptoms, which predominantly included dyspepsia, abdominal pain, and GORD-related symptoms (Table 3).

**Table 2:** Comparative Characteristics of All Cases of Celiac Disease Diagnosed During the Year 2010 in Two General Hospitals in Spain. Data are Alternatively Expressed as Mean ± Standard Deviation, or Number of Patient and Percentage

	Adult patients	Paediatric patients	p
Number or patients (%)	68 (86.1%)	11 (13.9%)	-
Female / Male (%)	50/18 (73.53%)	7/4 (63.64%)	0.49
Familiar association (%)	9 (13.2%)	3 (27.3%)	0.358
Positive tTGA (%)	13 (19.1%)	9 (81.8%)	0.004
Diagnostic delay (months)	70.67 ± 93	10.73 ± 12.77	< 0.001
Classic symptoms (%)	37 (54.4%)	10 (90.9%)	0.024
Haemoglobin levels (g/dL)	13.01 ± 2	12.02 ± 1.21	0.025
Serum Iron (µg/dL)	72.24 ± 45.43	58.20 ± 23.79	0.398
Ferritin (ng/mL)	53.16 ± 75.87	22.82 ± 9.92	0.245
Marsh I	45 (66.2%)	1 (9.1%)	< 0.001
Marsh II	10 (14.7%)	3 (27.3%)	0.38
Marsh III (a, b, c)	13 (19.1%)	7 (63.7%)	0.004
DQ2 haplotype	57 (85.1%)	8 (80%)	0.4
DQ8 haplotype	4 (6%)	2 (20%)	0.19
Non DQ2/DQ8	6 (9%)	0 (0%)	0.59

tTGA: anti-tissue transglutaminase antibodies; BMI: body mass index.

**Table 3:** Main Symptoms and Frequencies in Adult-onset CD at the Time of Diagnosi

	No (%)
Abdominal pain	29 (42.64 %)
Diarrhoea	25 (36.76 %)
Anaemia	16 (23.52 %)
Dyspepsia	11 (16.17 %)
GORD-related symptoms	10 (14.7 %)
Constipation	10 (14.7 %)
Vomiting	5 (7.35 %)
Weight loss	3 (4.41 %)
Dysphagia	3 (4.41 %)
Hypertransaminasemia	2 (2.94 %)
Hypocalcemia	1 (1.47 %)
Dermatitis	1 (1.47 %)

GORD: Gastroesophageal reflux disease.

Villous atrophy (Marsh III stages) in duodenal biopsies was present in 63.7% of the children and in only 19.1% of the adults (p = 0.004). Likewise, positive tTGA was present in 81.8% of the children as compared to only 19.1% of the adults (p = 0.004). Titters of tTGA were significantly different in relation to Marsh stages (P < 0.0005), with paired comparisons showing a significant difference between Marsh stages 3 and 1 (p < 0.0005) and Marsh stages 3 and 2 (p < 0.0005). Marsh stages 1 and 2 exhibited no significant differences in tTGA titters (p = 0.80), in good agreement with previously published findings [17-20]. As a probable consequence of a more severe enteropathy in paediatric patients, haemoglobin levels were significantly lower in children (p = 0.025), but no differences were observed regarding iron and ferritin blood levels.

## Discussion

This study analyzed all patients newly diagnosed with CD in two Spanish hospitals during the year 2010 and shows that in our current environment, the predominant diagnosis of the disease is in patients over the age of 16 (86.1%). This represents a relevant change in the epidemiology of what was traditionally considered a “children’s disease” to a problem affecting people of all ages, but with a clear predominance in adults. Several published papers had adverted from the 1980’s that the clinical picture of CD was already changing to milder forms, resulting in an upward shift of age at diagnosis, in such as way that the condition was not any more mainly a paediatric disorder [21, 22]. According to the recent literature, more than half of the diagnosed cases of CD currently occur in people aged 50 and older [8].

In our series, CD remains a predominantly female disease, with 72.2% of the cases appearing in women (63.63% < age 16 and 73.53% ≥ age 16). Adult CD presents a female/male ratio between 3:1 and 4:1 [5, 23], which is slightly higher than the well established 2:1 ratio found in children [24], and similar to previously reported results [21].

From a clinical point of view, manifestations of adult-onset CD are diverse and not necessarily related to the presence of a primary enteropathy. Only 25 adult patients in our study exhibited diarrhoea and 16 iron-deficiency anaemia, with most patients being diagnosed for atypical and extra-digestive symptoms, which are usually subclinical or of secondary importance. The first report which adverted about changes in clinical picture of CD also found that 55% of recent cases had no gastrointestinal symptoms; those few patients with diarrhoea were initially diagnosed as having irritable bowel syndrome because of not obvious malabsorption [21]. Even the classic image of a thin patient contrasts starkly with the average BMI results of our adult patients (24.67 kg/m^2^). Furthermore, 30 out of the 68 adult patients presented a BMI over 25, in agreement with the reported figures for overweight in up to 30% of adult celiac patients [12]. Reaching a diagnosis of CD in such patients usually takes several years, sometimes even decades, from the start of symptoms, which is significantly longer than for children. As a consequence, many adult celiac patients go undiagnosed for a longer time, with the average period of symptom evolution prior to diagnosis estimated to be between 3 and 17 years [5, 10, 13]. It should be noted that patients with active CD (clinically manifested) have a greater risk of death than the general population [8, 10], but that this normalizes 3 to 5 years after following a strict gluten-free diet (GFD) [8]. This data reinforces the benefit of maintaining a high level of suspicion and actively ruling out CD, even in patients with mild digestive manifestations and no diagnosis after initial studies.

CD in adults commonly represents a diagnostic challenge, since the classic symptoms of the disease contrast with their chameleonic clinical manifestations, the high frequency of negative tTGA titters, and the particularities found in the duodenal biopsies [7, 25, 26]. In this context, our research contains important data which should be carefully discussed.

The diagnosis of CD is relatively easy to achieve in cases presenting classic symptoms and unequivocal findings of small intestinal villous atrophy, but the complexity of adult-onset CD tends to require an active pursuit for a diagnosis. According to the European Society for Paediatric Gastroenterology, Hepatology, and Nutrition (ESPGHAN) [27], the study of all suspected patients, including children and adults, should begin with a blood test so that specific antibodies can be determined. Serological markers, especially IgA tTGA, are very useful indicators of CD since they identify the patients who need to undergo duodenal biopsies, which are still the diagnostic “gold standard.” However, this serological test has a significantly low sensitivity in most adult celiac patients; in fact, only 19.1% of adults included in our study presented positive titters. The test thus failed in identifying CD in the majority of cases in clinical practice. Taking this into account, an upper GI endoscopy for taking several duodenal biopsies should be always performed in all patients suspected of having CD, even in the face of negative serological results [25].

In 1992, Marsh presented an artificial classification of the spectrum of gluten enteropathy lesions observed in duodenal biopsies. These consisted of a series of several mucosal changes divided into four stages [3] presented as a continuous spectrum of small bowel mucosal deterioration due to gluten exposure. According to the ESPGHAN criteria [27], duodenal villous atrophy (Marsh stage III and onwards) is a criteria sine qua non for diagnosing celiac disease and only in this situation should a GFD be recommended. This assumption, which can be considered obsolete, was based on the traditional misinterpretation that Marsh I infiltrative lesions (also known as lymphocytic enteritis) were not associated with any symptom or sign of malabsorption [28]. In contrast, multiple findings have shown that villous intraepithelial lymphocytosis forms the most sensible histological index in many untreated adult CD patients [29-33], as well as in cases in which gluten is reintroduced after patients have adhered to a GFD or gluten challenge. Moreover, the majority of patients exhibiting dermatitis herpetiformis, an itchy blistering skin rash commonly associated with the ingestion of gluten and enteropathy, only exhibit the more subtle changes in duodenal biopsies consistent with the Marsh I stage [30, 34-36]. Still, great caution must be shown before diagnosing CD in patients presenting Marsh I lesions because intraepithelial lymphocytic infiltration represents a common, non-specific inflammatory response of the epithelium to a number of noxious or inflammatory signals. Many other conditions may show similar histological changes, such as peptic duodenitis, Helicobacter pylori infection, non-steroidal anti-inflammatory drug intake, autoimmune disorders, and parasitic infections [37]. CD has been estimated to be implied in only 10 to 16% of lymphocytic enteritis [32, 33]. Two recently published studies have shown that patients with lymphocytic enteritis can be categorized into two distinct groups: a) those patients presenting positive serology for CD and carrying CD-susceptible HLA haplotypes (who thus belong to the spectrum of gluten-sensitive enteropathy) and b) those who lack genetic and serological evidence of CD [38, 39].

Special consideration must be given to the diagnostic difficulties encountered in some so-called “borderline” mucosal findings. Villous tip analysis seems to be useful for distinguishing CD from non-specific changes early on, thus providing a valuable tool to use in routine clinical practice [40]. A limit of > 40 intraepithelial lymphocytes per 100 enterocytes was proposed to be abnormal in duodenal or jejunal mucosa by Ferguson and Murray in 1971 [41] before a lower threshold of 25% was determined by Hayat et al in 2002 [42]. Very recent research by Pellegrino et al concluded that the real threshold of duodenal intraepithelial lymphocytic infiltration may be even lower than that after determining that 25 intraepithelial lymphocytes per 100 enterocytes would miss 59% of CD cases after haematoxylin and eosin staining and 48% following CD3-staining [43]. As a last option, a CD diagnosis can be supported by an increased density of the gamma delta+ T cell receptor bearing intraepithelial lymphocytes in cases of borderline mucosal histology. This, however, requires frozen biopsy samples for analysis and the use of flow-cytometry for its identification [29]. In any case, the diagnosis of CD must be based on firm evidence since a life-long GFD is expensive and difficult to maintain for many individuals, especially because of the many social restrictions it entails. For these reasons, additional features should always be actively sought out.

Regarding serological markers, it should be noted that the tTGA titters correlate linearly with the stage of the histological lesion, with a higher sensitivity and specificity for CD diagnosis in patients with villous atrophy [44-46], a finding corroborated in our study. All paediatric and adult patients in our series with high tTGA titters presented Marsh III stages in duodenal biopsies, while most patients with negative titters exhibited Marsh I stages. These data are in concordance with the available information showing that tTGA sensitivity is very low in children in whom only lymphocytic enteritis is observed [45, 46]. In adults, on the other hand, it is the most common finding, representing 66.2% of our adult study subjects. Overall the sensitivity for tTGA has been estimated to exceed 90% in childhood cases, but is reduced to a mere 15 - 30%, approximately, in adults [19]. The well-known low sensitivity of tTGA in diagnosing adult-onset CD also limits its use in population screening. Recent studies aiming to estimate the prevalence of CD through serological screening [24] have found that incidence of CD was higher in children than in adults, a biased finding that clearly underestimates the current reality of the disease. As we have shown, negative tTGA results do not definitively rule out a diagnosis of CD (as happened in 81.9% of our adult celiac patients, but only in 18.2% of the children) since it is very common for adults to have negative serology. This is the strongest argument for performing a genetic study, which is necessary if there is very strong suspicion of CD because it has a very high negative predictive value.

Genetic studies can also be undertaken prior to carrying out the definitive but invasive diagnosis by means of duodenal biopsies. A strategy based on the genetic study of first-degree relatives followed by duodenal biopsies in positive cases diagnosed 3 times the number of cases than serology alone [47], mostly because of the high proportion of symptomatic patients with lymphocytic enteritis (Marsh I) in the adult celiac population. CD has a higher class II MHC association than detected previously in many other autoimmune diseases [48]. Indeed, approximately 90% of celiac patients carry the HLA-DQ2 heterodimer, which is common to many autoimmune diseases. According to these figures, 81.81% of the children and 85.3% of the adults diagnosed with CD in one year at our hospitals expressed a DQ2 haplotype. Most patients who were DQ2 negative carried the DQ8 genotype, as did 18.18% of the children and 5.88% of adults in our series. In the last few years, genome-wide association studies (GWAS) performed in large numbers of CD patients, relatives, and match controls have revealed evidence of additional non-HLA loci of CD susceptibility, most of which are related to T-cell regulation and inflammation [1, 49]. These studies will allow us to understand why only a small number of individuals who carry these alleles, which are expressed in up to 25% of the general population, develop CD. They will also help us characterize clearly as CD sufferers the small number of patients who are both DQ2 and DQ8 negative, as observed in 8.82% of the adult patients in our series and which has been described to occur in 6% of the European population [50]. In fact, a recent study evaluating the frequency of DQ2 and DQ8 alleles in 127 consecutive cases of adult-onset CD found that all patients with atypical HLA responded to a gluten-free diet [51]. Most DQ2 and DQ8 negative patients in our series encoded half of the DQ2 heterodimer as the low-risk haplotype DQ2.02 (DQA1*02 and DQB1*02) or as DQA*05, as recently described in case series from Europe [50] and the USA [51] for patients who responded to a GFD.

In this context, different CD experts agree that because of the difficulty of establishing a diagnosis through other tests, the clinical and histological response to a strict GFD for at least 6 months represents the most definitive diagnostic test, particularly when accompanied by improvement or normalization of the previously altered laboratory test parameters with no concomitant medication [25, 29]. Such experts do not recommend the repetition of duodenal biopsies following gluten reintroduction in the case of adults with sufficient diagnostic criteria and a good response to the prescribed diet; however gluten sensitivity, a newly identified disease recently added to the spectrum of gluten related disorders [14, 15], can not ruled out if histological recover is not assayed in duodenal biopsies. This new disorder represents an important clinical entity which is not linked to autoimmune co-morbidity and to risk of severe complications, such as small bowel lymphoma. The response to a GFD is clearly positive in gluten sensitivity, but a positive serological test, mucosal alterations and altered biochemical nutritional markers use to be absent [14, 15].

Taken together, these findings lead us to the conclusion that current diagnostic algorithms of CD [27] are not useful in adult-onset cases. In fact, a recent survey conducted by ESPGHAN concluded that a revision of criteria for diagnosing CD is urgently needed [52].

In this sense, screening of adult-onset CD through tTGA titters should be abandoned due to its low sensitivity, which limits the number of diagnosed cases and underestimates the real prevalence in this age group. The only advantage of tTGA in this age group comes from its high positive predictive value, but duodenal biopsy cannot be avoided, since it is necessary to confirm the diagnosis. The presence of a high-risk HLA haplotype is not a diagnostic criterion per se, but if DQ2 or DQ8 are positive, the pre-biopsy probability increases. The current concept that villous atrophy is required for prescription of a gluten-free diet should be updated, since most adult patients only present lymphocytic epithelial infiltration [36] and, consequently, negative tTGA titters, but their symptoms, quality of life, and extra-digestive associated diseases clearly improve after excluding gluten from the diet.

Only a rupture with the classic paradigm of the celiac patient and maintaining a high level of suspicion in multiple medical scenarios will allow us to properly treat the great proportion of celiac patients that remains undiagnosed twenty centuries after Aretaeus of Cappadocia first described the disease.

## References

[1] Schuppan D, Junker Y, Barisani D (2009). Celiac disease: from pathogenesis to novel therapies. Gastroenterology.

[2] Trier JS (1991). Celiac sprue. N Engl J Med.

[3] Marsh MN (1992). Gluten, major histocompatibility complex, and the small intestine. A molecular and immunobiologic approach to the spectrum of gluten sensitivity (‘celiac sprue'). Gastroenterology.

[4] Sundar N, Crimmins R, Swift G (2007). Clinical presentation and incidence of complications in patients with coeliac disease diagnosed by relative screening. Postgrad Med J.

[5] Fernandez A, Gonzalez L, de-la-Fuente J (2010). Coeliac disease: clinical features in adult populations. Rev Esp Enferm Dig.

[6] Rodrigo-Saez L, Fuentes-Alvarez D, Perez-Martinez I, Alvarez-Mieres N, Nino-Garcia P, de-Francisco-Garcia R, Riestra-Menendez S (2011). Differences between pediatric and adult celiac disease. Rev Esp Enferm Dig.

[7] Fernandez Salazar LI, de la Torre Ferrera N, Velayos Jimenez B, Nocito Colon M, Gonzalez Hernandez JM, Garrote Adrados JA (2008). Diagnostic problems in adult celiac disease. Rev Esp Enferm Dig.

[8] Goddard CJ, Gillett HR (2006). Complications of coeliac disease: are all patients at risk?. Postgrad Med J.

[9] Green PH, Cellier C (2007). Celiac disease. N Engl J Med.

[10] Gasbarrini G, Ciccocioppo R, De Vitis I, Corazza GR (2001). Coeliac Disease in the Elderly. A multicentre Italian study. Gerontology.

[11] Rewers M (2005). Epidemiology of celiac disease: what are the prevalence, incidence, and progression of celiac disease?. Gastroenterology.

[12] Garcia-Manzanares A, Lucendo AJ (2011). Nutritional and dietary aspects of celiac disease. Nutr Clin Pract.

[13] (2004). NIH Consensus Development Conference on Celiac Disease. NIH Consens State Sci Statements.

[14] Troncone R, Jabri B (2011). Coeliac disease and gluten sensitivity. J Intern Med.

[15] Sapone A, Lammers KM, Casolaro V, Cammarota M, Giuliano MT, De Rosa M, Stefanile R (2011). Divergence of gut permeability and mucosal immune gene expression in two gluten-associated conditions: celiac disease and gluten sensitivity. BMC Med.

[16] Farrell RJ, Kelly CP (2002). Celiac sprue. N Engl J Med.

[17] Fasano A, Berti I, Gerarduzzi T, Not T, Colletti RB, Drago S, Elitsur Y (2003). Prevalence of celiac disease in at-risk and not-at-risk groups in the United States: a large multicenter study. Arch Intern Med.

[18] Murray JA (2005). Celiac disease in patients with an affected member, type 1 diabetes, iron-deficiency, or osteoporosis?. Gastroenterology.

[19] Hill PG, McMillan SA (2006). Anti-tissue transglutaminase antibodies and their role in the investigation of coeliac disease. Ann Clin Biochem.

[20] Dickey W, Hughes D (2001). Disappointing sensitivity of endoscopic markers for villous atrophy in a high-risk population: implications for celiac disease diagnosis during routine endoscopy. Am J Gastroenterol.

[21] Logan RF, Tucker G, Rifkind EA, Heading RC, Ferguson A (1983). Changes in clinical features of coeliac disease in adults in Edinburgh and the Lothians 1960-79. Br Med J (Clin Res Ed).

[22] Maki M, Kallonen K, Lahdeaho ML, Visakorpi JK (1988). Changing pattern of childhood coeliac disease in Finland. Acta Paediatr Scand.

[23] Llorente-Alonso MJ, Fernandez-Acenero MJ, Sebastian M (2006). Gluten intolerance: sex and age-related features. Can J Gastroenterol.

[24] Marine M, Farre C, Alsina M, Vilar P, Cortijo M, Salas A, Fernandez-Banares F (2011). The prevalence of coeliac disease is significantly higher in children compared with adults. Aliment Pharmacol Ther.

[25] Rodrigo L (2006). Celiac disease. World J Gastroenterol.

[26] Lucendo Villarin AJ, Martin Plaza J, Comas Redondo C (2006). Celiac disease in adult patients. A different clinical spectre. An Med Interna.

[27] Revised criteria for diagnosis of coeliac disease (1990). Report of Working Group of European Society of Paediatric Gastroenterology and Nutrition. Arch Dis Child.

[28] Ciclitira PJ, King AL, Fraser JS (2001). AGA technical review on Celiac Sprue. American Gastroenterological Association. Gastroenterology.

[29] Collin P, Wahab PJ, Murray JA (2005). Intraepithelial lymphocytes and coeliac disease. Best Pract Res Clin Gastroenterol.

[30] Ferguson A, Arranz E, O'Mahony S (1993). Spectrum of expression of intestinal cellular immunity: proposal for a change in diagnostic criteria of celiac disease. Ann Allergy.

[31] Arranz E, Ferguson A (1993). Intestinal antibody pattern of celiac disease: occurrence in patients with normal jejunal biopsy histology. Gastroenterology.

[32] Kakar S, Nehra V, Murray JA, Dayharsh GA, Burgart LJ (2003). Significance of intraepithelial lymphocytosis in small bowel biopsy samples with normal mucosal architecture. Am J Gastroenterol.

[33] Goldstein NS, Underhill J (2001). Morphologic features suggestive of gluten sensitivity in architecturally normal duodenal biopsy specimens. Am J Clin Pathol.

[34] Ferguson A, Blackwell JN, Barnetson RS (1987). Effects of additional dietary gluten on the small-intestinal mucosa of volunteers and of patients with dermatitis herpetiformis. Scand J Gastroenterol.

[35] Reunala T (1998). Dermatitis herpetiformis: coeliac disease of the skin. Ann Med.

[36] Kaukinen K, Maki M, Partanen J, Sievanen H, Collin P (2001). Celiac disease without villous atrophy: revision of criteria called for. Dig Dis Sci.

[37] Aziz I, Evans KE, Hopper AD, Smillie DM, Sanders DS (2010). A prospective study into the aetiology of lymphocytic duodenosis. Aliment Pharmacol Ther.

[38] Vande Voort JL, Murray JA, Lahr BD, Van Dyke CT, Kroning CM, Moore SB, Wu TT (2009). Lymphocytic duodenosis and the spectrum of celiac disease. Am J Gastroenterol.

[39] Kurppa K, Collin P, Viljamaa M, Haimila K, Saavalainen P, Partanen J, Laurila K (2009). Diagnosing mild enteropathy celiac disease: a randomized, controlled clinical study. Gastroenterology.

[40] Jarvinen TT, Collin P, Rasmussen M, Kyronpalo S, Maki M, Partanen J, Reunala T (2004). Villous tip intraepithelial lymphocytes as markers of early-stage coeliac disease. Scand J Gastroenterol.

[41] Ferguson A, Murray D (1971). Quantitation of intraepithelial lymphocytes in human jejunum. Gut.

[42] Hayat M, Cairns A, Dixon MF, O'Mahony S (2002). Quantitation of intraepithelial lymphocytes in human duodenum: what is normal?. J Clin Pathol.

[43] Pellegrino S, Villanacci V, Sansotta N, Scarfi R, Bassotti G, Vieni G, Princiotta A (2011). Redefining the intraepithelial lymphocytes threshold to diagnose gluten sensitivity in patients with architecturally normal duodenal histology. Aliment Pharmacol Ther.

[44] Hill ID (2005). What are the sensitivity and specificity of serologic tests for celiac disease? Do sensitivity and specificity vary in different populations?. Gastroenterology.

[45] Tursi A, Brandimarte G, Giorgetti GM (2003). Prevalence of antitissue transglutaminase antibodies in different degrees of intestinal damage in celiac disease. J Clin Gastroenterol.

[46] Rostom A, Dube C, Cranney A, Saloojee N, Sy R, Garritty C, Sampson M (2005). The diagnostic accuracy of serologic tests for celiac disease: a systematic review. Gastroenterology.

[47] Esteve M, Rosinach M, Fernandez-Banares F, Farre C, Salas A, Alsina M, Vilar P (2006). Spectrum of gluten-sensitive enteropathy in first-degree relatives of patients with coeliac disease: clinical relevance of lymphocytic enteritis. Gut.

[48] Thorsby E, Lie BA (2005). HLA associated genetic predisposition to autoimmune diseases: Genes involved and possible mechanisms. Transpl Immunol.

[49] Romanos J, van Diemen CC, Nolte IM, Trynka G, Zhernakova A, Fu J, Bardella MT (2009). Analysis of HLA and non-HLA alleles can identify individuals at high risk for celiac disease. Gastroenterology.

[50] Karell K, Louka AS, Moodie SJ, Ascher H, Clot F, Greco L, Ciclitira PJ (2003). HLA types in celiac disease patients not carrying the DQA1*05-DQB1*02 (DQ2) heterodimer: results from the European Genetics Cluster on Celiac Disease. Hum Immunol.

[51] Harmon GS, Lebeck LK, Weidner N (2011). Gluten-dependent enteropathy and atypical human leukocyte antigen alleles. Hum Pathol.

[52] Ribes-Koninckx C, Mearin M, Korponay-Szabo I, Shamir R, Husby S, Ventura A, Branski D (2011). Coeliac disease diagnosis: espghan 1990 Criteria or need for a change? Results of a questionnaire. J Pediatr Gastroenterol Nutr.

